# Analysis of vegetation coverage changes and driving forces in the source region of the yellow river

**DOI:** 10.1038/s41598-025-06921-x

**Published:** 2025-07-02

**Authors:** Kaining Yu, Caijia Yang, Tao Wu, Yifeng Zhai, Shixiong Tian, Yuqing Feng

**Affiliations:** 1 Hebei Center for Ecological and Environmental Geology Research, Hebei GEO University, Shijiazhuang, 050031 China; 2 School of Water Resources and Environment, Hebei GEO University, Shijiazhuang, 050031 China; 3 Hebei Key Laboratory of Geological Resources and Environment Monitoring and Protection, Hebei Geo-Environment Monitoring, Shijiazhuang, 050031 China; 4 HeBei Branch of China National Geological Exploration Center of Building Materials Industry, Baoding, 071000 China

**Keywords:** Temporal mutation test, Normalized vegetation index (NDVI), The source region of the yellow river, Spatial and Temporal variability, Driving factors, Ecology, Climate-change ecology

## Abstract

As a significant ecological barrier, the source region of the Yellow River serves as a crucial water source in China, and its vegetation dynamics play a pivotal role in water conservation. Monitoring vegetation dynamics is essential for ecological protection and the achievement of sustainable development goals, as it facilitates systematic assessment of vegetation restoration, supports the advancement of ecological civilization, and promotes coordinated economic and environmental development. Based on the newly released AVHRR GIMMS NDVI3g data from 1982 to 2020 provided by the NASA Goddard Space Flight Center, this study aims to identify the driving mechanisms influencing vegetation dynamics in the source region of the Yellow River over the past 40 years (1982–2020) by utilizing ordered cluster analysis, Pearson correlation analysis, and the Geodetector method. The influence of each driving factor on NDVI was systematically examined, and the spatial and temporal characteristics of vegetation as well as the effects of key drivers were clarified to inform ecological protection and sustainable development strategies. The results indicate that: (1) the overall NDVI in the source region of the Yellow River exhibited a significant upward trend from 1982 to 2020, with a noticeable shift occurring in 2009. Prior to 2009, NDVI demonstrated a slight declining trend, whereas a significant increase was observed afterward; (2) NDVI distribution displayed a spatial gradient, increasing from northwest to southeast, with higher values in the southeast and lower values in the northwest; (3) the interaction between any two driving factors had a more substantial influence on NDVI than individual factors, demonstrating a two-factor enhancement effect. Notably, the interaction between precipitation and temperature with other variables exhibited the strongest explanatory power, with q-values exceeding 0.5. Overall, natural factors such as temperature and precipitation played a crucial role in NDVI variation, and the abrupt change in 2009 may be attributed to regional warming and the implementation of ecological protection measures.

## Introduction

 Amid global warming, significant alterations in regional hydrological cycles and moisture conditions have profoundly impacted global vegetation growth and development^1^. Vegetation is pivotal in terrestrial ecosystems, serving as a natural interface among the atmosphere, soil, and water^2^. Vegetative cover often reflects the quality of the ecological environment. The Normalized Difference Vegetation Index (NDVI) is a widely used remote sensing metric that characterizes vegetation cover and effectively indicates large-scale surface vegetation health and growth^3,4^.

Numerous studies utilizing remote sensing vegetation monitoring have been conducted on the source region of the Yellow River, with vegetation analysis becoming increasingly prevalent in recent years. Analysis of vegetation change and climate impacts in the source region of the Yellow River reveals that precipitation and radiation are the primary climatic drivers influencing the vegetation index of the Yellow River Basin^23^; The spatial distribution of NDVI in the Yellow River Basin aligns with the availability of water and heat resources; The climate of the source region of the Yellow River has gradually changed from dry and cold to warm and humid, and the vegetation cover shows the trend of overall slow increase and local degradation. Vegetation improvement in the Yellow River source is predominantly observed south of Zaling Lake and Eling Lake, while degradation primarily occurs north of these lakes, with vegetation changes being more sensitive to temperature^17–19^; Temperature, land surface temperature, and precipitation in the source region of the Yellow River exhibited an upward trend from 1982 to 2014, with a notable shift in 2007 marking a transition from a “dry” to “humid” climate state. In this climatic context, the tundra environment has degraded, while vegetation NDVI has shown a slight increase^20^. In recent years, climate warming and humidification in the Tibetan Plateau have become well-established, with changes in the climate environment and the implementation of the Sanjiangyuan Ecological Protection Project being the primary drivers of the overall improvement in the ecological function of Sanjiangyuan National Park^21^.

Previous studies can reflect the characteristics of vegetation cover changes in the source region of the Yellow River to a certain extent, but they generally focus on the overall characteristics of vegetation cover changes, and do not give much consideration to the characteristics of mutations occurring in the process of vegetation changes; however, the information of mutations in the process of vegetation evolution can be used to reveal the ecosystem changes, assess the quality of the ecological environment, warn of natural disasters, and support the policy formulation and adjustment. The study in this paper, however, identifies the mutation points through ordered cluster analysis to reveal the stage characteristics of vegetation dynamic evolution, and then segments to explore the relationship between NDVI and driving factors before and after the mutation, which makes up for the neglect of nonlinear mutation in traditional trend analysis. A geodetector model is introduced to quantify the contributions of natural factors such as precipitation and temperature and their interactions to the spatial differentiation of NDVI, which deepens the understanding of the complex driving mechanism of alpine ecosystems, and provides a scientific basis for the assessment of the effects of ecological restoration policies.

## Materials and methods

### Study area

**Fig. 1:**
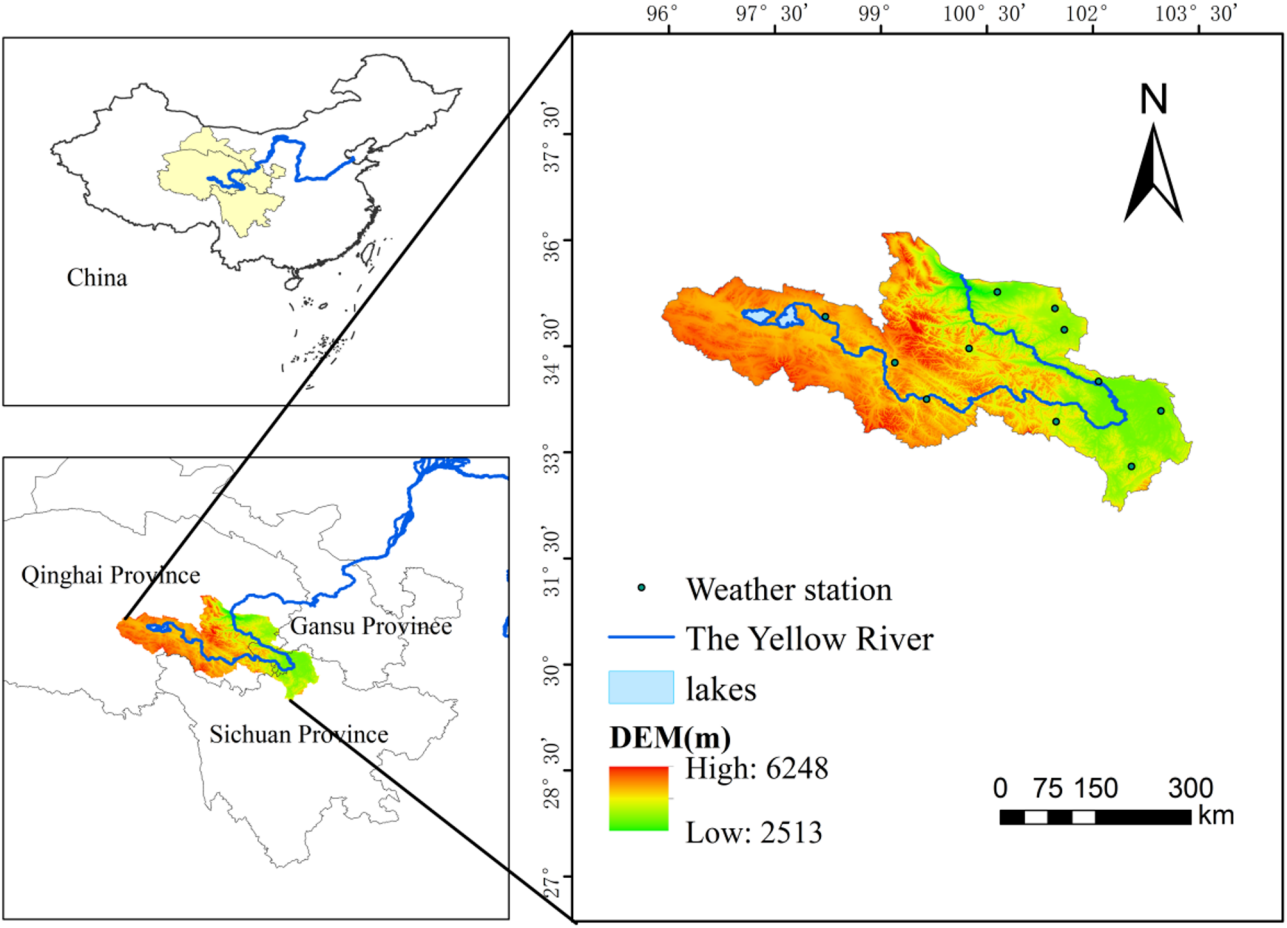
Geographical location and distribution of meteorological stations in the source region of the Yellow River (Cartographic software: ArcGIS 10.6, https://desktop.arcgis.com/).

The source region of the Yellow River (one of the three sources of the river): it refers to the Yellow River basin above Longyangxia Hydropower Station, located in the northeast of the Tibetan Plateau in the scope of the Yellow River basin, with a geographic location of 32°12′−36°36’N, 95°54–103°24’E, involving 6 prefectures and 18 counties in Qinghai, Sichuan and Gansu provinces, with a total area of about 132,000 km^2^ (Fig. 1). The overall topography of the source region of the Yellow River is characterized by high northwest and low southeast, with great undulations. The area features high mountains, canyons, basins, glaciers, and other landforms, interspersed with numerous rivers and lakes. The altitude range of the area is 2513–6248 m, with an average elevation of 4300 m. The source region of the Yellow River generally experiences a continental plateau climate^22^: cold and dry, with long winters, short summers, large diurnal temperature variations, strong solar radiation, monsoon influence, and precipitation mainly concentrated between June and September. The vegetation in the source region the Yellow River exhibits typical characteristics of an alpine ecosystem, with its types and spatial distribution patterns primarily regulated by hydrothermal conditions, topography, and the presence of permafrost. Four major vegetation types are found in the region. Alpine meadow, the dominant vegetation type, covers approximately 60–85% of the area. Alpine grassland is concentrated in the arid northwestern region at elevations of 3600–4500 m, with a coverage of 30–50%. Alpine thicket primarily occurs in the southeastern river valleys at elevations between 3200 and 4200 m. Swampy meadow is mainly distributed in the river source depressions at elevations of 3200–3800 m, with a coverage of more than 90%. In terms of spatial distribution, the vegetation exhibits significant vertical zonation and a distinct horizontal gradient. Vertically, with increasing elevation, vegetation transitions from scrub to meadow, then to grassland, and eventually to sparse vegetation. Horizontally, from southeast to northwest, the vegetation transitions from dense meadow and scrub to moderately dense grassland and finally to sparse desert grassland, a pattern that is strongly correlated with the regional gradient of annual precipitation.

### Data sources and pre-processing

This study aimed to analyze vegetation growth trends ithe source region of the Yellow River from 1982 to 2020 using the AVHRR GIMMS NDVI3g dataset (15-day composite period, 0.083° × 0.083° spatial resolution) provided by NASA Goddard Space Center (https://ecosystem.nasa.gov/). The NDVI dataset was formed through data preprocessing (subset extraction, mosaicing, cropping, format conversion, projection conversion, quality checks, etc.), and the maximum value compositing (MVC) method was used to extract the average of the maximum NDVI values between May and September to characterize the vegetation growth. Additionally, meteorological data (precipitation, air temperature, etc.) from ERA5-Land (https://cds.climate.copernicus.eu/), land-use data (2020) from the Remote Sensing Monitoring Database of China’s Land Use Status (RSMDC) (10.5281/zenodo.8176941), the ASTER GDEM V3 elevation data (30 m resolution; http://www.gscloud.cn/), GDP data from the National Earth System Science Data Center (http://www.geodata.cn/), and population density data from the Resource and Environmental Science Data Platform (http://www.resdc.cn/) were utilized for watershed analysis (Table 1). The watershed boundary of the source region of the Yellow River was delineated by splicing, processing flow direction, and integrating river network data. To ensure spatial consistency and meet the analytical requirements of Geodetector this study systematically preprocessed all datasets using the ArcGIS 10.6 platform through uniform cropping and coordinate conversion, and standardized them to a consistent spatial resolution using the resampling tool.

**Table 1:** Data Overview.

Name	Source	Format	Resolution	Time/year
NDVI	AVHRR GIMMS NDVI3g	.tif	0.083°×0.083°	1982–2020
Precipitation(P)	ERA5-land	.tif	0.1°	1982–2020
Temperature(T)	ERA5-land	.tif	0.1°	1982–2020
Soil moisture(SM)	ERA5-land	.tif	0.1°	1982–2020
Soil temperature(ST)	ERA5-land	.tif	0.1°	1982–2020
Actual evapotranspiration(ETA)	ERA5-land	.tif	0.1°	1982–2020
DEM(elevation)	Geospatial data cloud	.tif	30 m	2020
Land-use type	China Land Use Status Remote Sensing Monitoring Database	.tif	1 km	2020
Slope				2020
Slope aspect				2020
GDP	National Earth System Science Data Center	.tif	1 km	2020
Population density	Resource and Environmental Science Data Platform	.tif	100 m	2020

## Research methods

### NDVI classification

The pre-processed NDVI images were used to mitigate the effects of outliers using the MVC method^4^. Based on existing studies^5–10^the annual maximum NDVI in the area was classified into six classes using the equal interval reclassification method to study the spatial and temporal variations of vegetation cover (see Table 2).

**Table 2:** Classification criteria for vegetation cover status based on normalized vegetation index (NDVI).

Overlay partition	NDVI
Bare area	⩽0
Low coverage area	0 ~ 0.2
Low and medium coverage area	0.2 ~ 0.4
Medium coverage area	0.4 ~ 0.6
Medium and high coverage area	0.6 ~ 0.8
High coverage area	0.8 ~ 1

### Trend analysis

To visually characterize the change trend of vegetation cover in the area, using images processed by the MVC method, a study was conducted on the vegetation cover change trend between 1982 and 2020, employing univariate linear regression analysis. Univariate linear regression predicts the change trend by analyzing temporal variables. The calculation is based on the following formula: (1)

1 $${\theta _{{\text{slope}}}}=\frac{{n \times \sum\limits_{{i=1}}^{n} {(i \times NDV{I_i}) - } (\sum\limits_{{i=1}}^{n} i ) \times (\sum\limits_{{i=1}}^{n} {NDVI_i} )}}{{n \times \sum\limits_{{i=1}}^{n} {{i^2} - (} \sum\limits_{{i=1}}^{n} i )2}}$$

where: *θ*_slope_ represents the regression slope of the univariate linear equation, *n* denotes the cumulative number of years in the monitoring period (*n* = 39), and *NDVI*_*i*_ refers to the NDVI value in the *i*th year. The significance of interannual variability was assessed by analyzing the correlation between vegetation cover and the time series. A positive regression slope indicates an increase in vegetation cover, while a negative value indicates a decrease. To accurately analyze changes in vegetation cover in the region, *θ*_slope_ values were classified into five categories, with the corresponding trends in vegetation cover changes presented in Table 3.

**Table 3:** Classification criteria for trends in vegetation cover change based on the slope (*θ*_slope)_ of the regression of the univariate linear equation for vegetation cover change.

Level	θ_slope_
Obvious degradation	≤−0.0019
Mildde gradation	−0.0019~−0.0010
Basically stable	−0.0010~−0.0002
Slight improvement	−0.0002 ~ 0.0006
Significant improvement	≥ 0.0006

### Correlation calculations

In the present study, Pearson’s correlation coefficient was used to characterize the correlation between NDVI and meteorological factors such as temperature and precipitation^12^ which was calculated as follows.

2 $${R_{xy}}=\frac{{\sum\limits_{{i=1}}^{n} {[({x_i} - \overline {x} )({y_i} - \overline {y} )]} }}{{\sqrt {\sum\limits_{{i=1}}^{n} {{{({x_i} - \overline {x} )}^2}} \sum\limits_{{i=1}}^{n} {{{({y_i} - \overline {y} )}^2}} } }}$$

where, *R*_*xy*_ represents the correlation coefficient between the two variables, ranging from 1 to −1. A value of 1 indicates a perfect positive correlation, 0 indicates no correlation, and − 1 indicates a perfect negative correlation. Here, *x*_*i*_ and *y*_*i*_ represent the vegetation cover and meteorological factors for the *i*th year, respectively, while *x* and *y* denote their multi-year averages. Additionally, *i* represents the number of samples.

### The geodetector method

Geodetector is a statistical method whose core idea is that the spatial distributions of an independent variable and a dependent variable should have similarity if an independent variable has an important effect on the dependent variable. In this study, factor detection and interaction detection were used to analyse the spatial differentiation of vegetation NDVI in the source region of the Yellow River.

The specific model is as follows:

1) Factor Detection: It is used to detect the spatial variability of vegetation NDVI (Y) in the study area and the magnitude of the explanatory power of the detection factor (X) on the spatial variability of its vegetation NDVI (Y), measured by q-value:

3 $$q=1-\frac{{\sum\limits_{{h=1}}^{L} {{N_h}{\delta _h}^{2}} }}{{{N_\delta }^{2}}}$$

In the equation, *q* represents the percentage of variability in Y explained by the independent variable X, where 100 × *q*% of Y is explained by X. *h* = 1, 2, …, *L* represents the strata of variable Y or factor X; *N*_*h*_ and *N* are the number of units in stratum *h* and the entire area, respectively; *δ²*_*h*_ and *δ²* are the variances of Y in stratum *h* and the entire area, respectively.

2) Interaction detection: This method is used to identify the degree of influence that the interaction between different natural geographical factors has on the vegetation NDVI in the study area. Based on the q values of any two factors (A, B) and the interaction q value of the two factors (C), the results are classified into five categories: nonlinear weakening (C < min(A, B)),single-factor nonlinear weakening (min(A, B) < C < max(A, B)), double-factor enhancement (C > max(A, B)), independence (C = A + B), and nonlinear enhancement (C > A + B).

In this study, eleven natural geographic factors: precipitation (X1), temperature (X2), actual evapotranspiration (X3), soil moisture (X4), soil temperature (X5), elevation (X6), slope (X7), slope aspect (X8), land-use type (X9), GDP (X10) and population density (X11) were selected to explore their effects on the changes in vegetation NDVI (Y) in the source region of the Yellow River. Based on the principle of the Geodetector, the numerical independent variables were discretized, and the natural breakpoint method was used to classify the above factors. In ArcGIS 10.6, the Create Fishnet tool was used to generate 1678 random sampling points (57 outliers were removed), and the attribute values of the annual average vegetation NDVI with the nine geographic factors were extracted to these sampling points by the Multiple Extraction to Points and Resampling tools to obtain the corresponding data of the annual average vegetation NDVI with each natural geographic factor in the source region of the Yellow River from 1982 to 2020.

### Mutation analysis and mutation inflection point identification

#### Ordered cluster analysis (OCA)

Ordered cluster analysis is a statistical method used for time series data. Its core objective is to identify the optimal split point (also referred to as a mutation point), such that the sum of squared deviations within similar classes is minimized, and the sum of squared deviations between different classes is maximized. The mean values of the time series before and after the mutation point are then computed.

4 $${V_\tau }{\text{ =}}\sum {{{{\text{(}}{X_i} - {{\overline {X} }_\tau }{\text{)}}}^2}}$$

5 $${V_{n-\tau }}{\text{ =}}\sum {{\text{(}}{X_i}{\text{ }} - {{\overline {X} }_{_{{n-\tau }}}}{\text{)}}{{\text{ }}^2}}$$

6 $${\overline {x} _{_{\tau }}}=\frac{1}{\tau }\sum\limits_{{t=1}}^{\tau } {{x_t}}$$

7 $${\overline {x} _{_{{n - \tau }}}}=\frac{1}{{n-\tau }}\sum\limits_{{t=\tau +1}}^{n} {{x_t}}$$

Total sum of squared deviations

8 $$S_n (\tau) =V_{\tau} +V_{n-\tau}$$

where: *τ* is the potential mutation point, ‾*X*_*τ*_ and ‾*X*_*n−τ*_ represent the mean values of the sequences before and after the mutation point, respectively; *V*_*τ*_ and *V*_*n−τ*_ denote the sums of squared deviations for the two segments of the sequence; and *Sn(τ)* is the sum of squared deviations at point *τ*.

Based on the total sum of deviations, find the optimal split point, which is calculated as:

9 $$S^*_n = \min_{{-1 \leq \tau \leq 1}}\{S_n(\tau) \}$$

The value of *τ* that satisfies the above equation represents the optimal splitting point, corresponding to the mutation time.

Generally speaking, if the time series exhibits two distinct stages, the total sum of squared deviations will display a single valley. If there are two or more distinct stages, the total sum of squared deviations will exhibit multiple valleys, allowing the identification of phase transitions in the sequence based on the timing of these valleys.

**Fig. 2:**
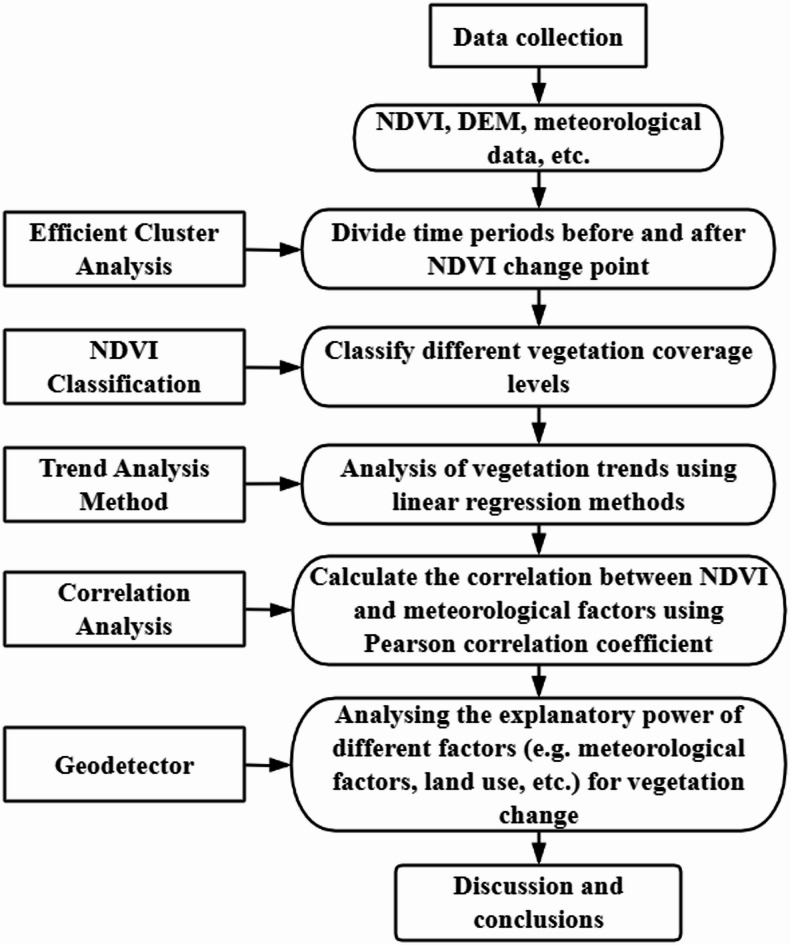
Flowchart of Research Methods.

Figure 2 shows the flowchart of research methods, which demonstrates the research idea of this paper. Taking the source region of the Yellow River as the research object, based on NDVI and climate data from 1982 to 2020, the ordered cluster analysis method is utilized to test the mutation of NDVI in the region, so as to discuss the linkage between NDVI and related factors before and after the year of mutation in a segmented manner.

## Results and analyses

### Analysis of NDVI mutation points

**Fig. 3:**
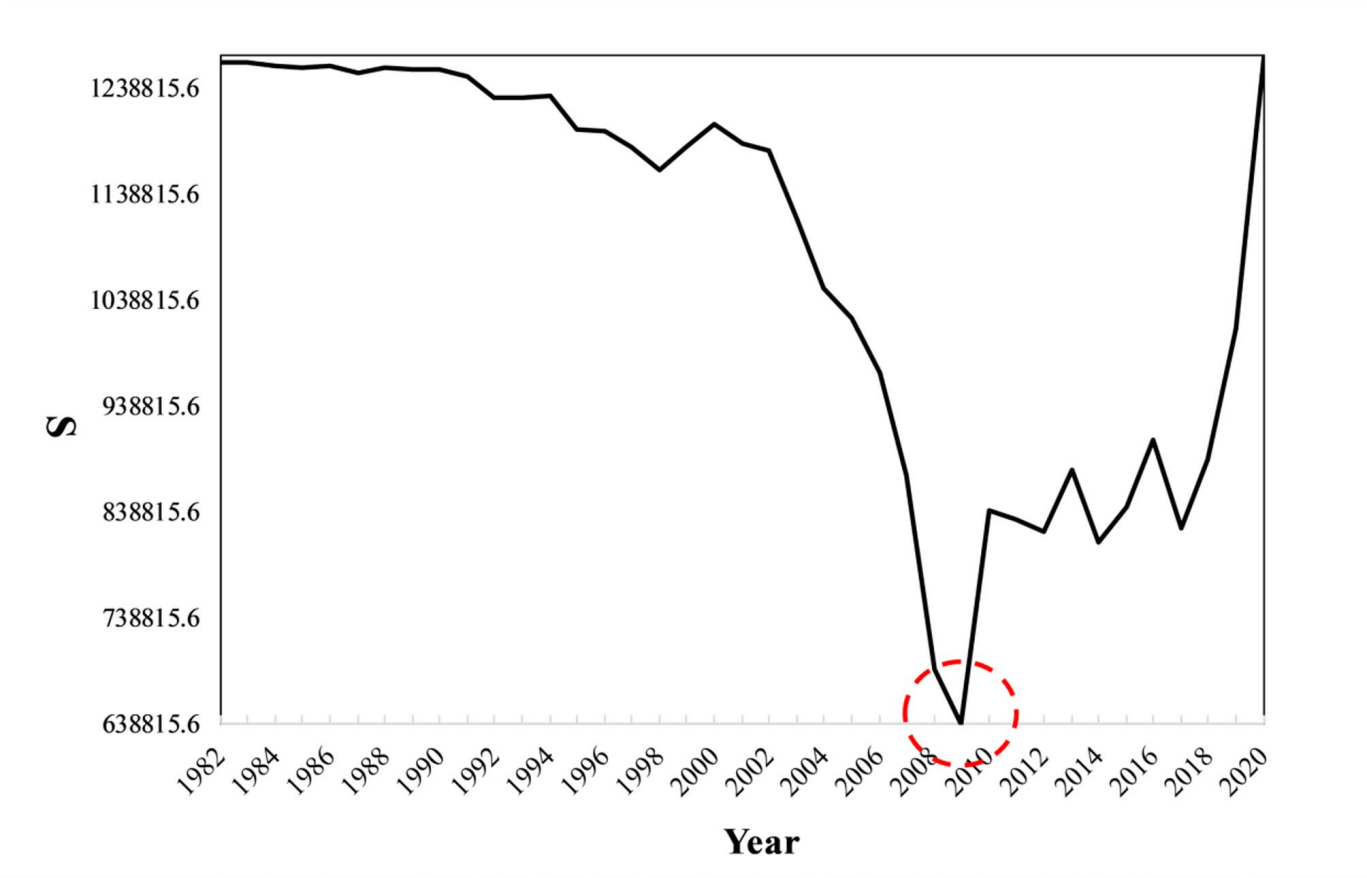
NDVI ordered cluster mutation analysis.

Using the ordered cluster analysis method to analyze the NDVI data of the source region of the Yellow River, the corresponding Sn(τ) for τ was calculated, and the NDVI change curve was plotted, as shown in the figure below (Fig. 3). From the figure, it can be observed that 2009 serves as the change point. The ordered cluster test value |T|=5.82 > T (0.05/2) = 1.64, indicating that there was a significant jump in the mean values before and after 2009. Using 2009 as the dividing point, the NDVI data for the source region of the Yellow River were divided into two periods: 1982–2009 and 2010–2020.

### Characteristics of temporal changes in vegetation cover

**Fig. 4:**
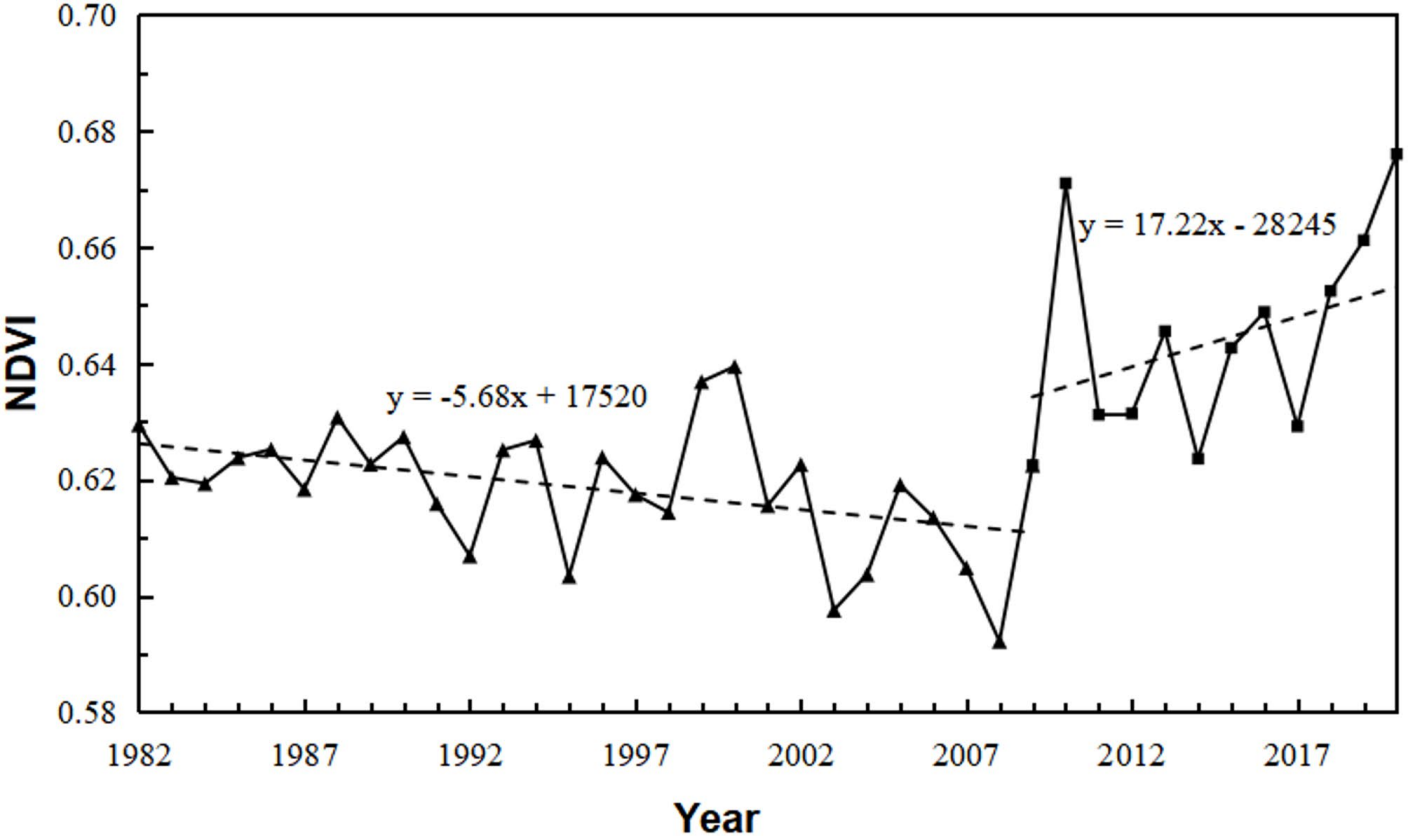
Trend of NDVI changes in the source region of the Yellow River in May-September months of each year from 1982 to 2020.

Utilizing the Maximum Value Composite (MVC) method, the annual NDVI data for the source region of the Yellow River were derived by selecting the maximum NDVI values from May to September for each year between 1982 and 2020^11^. The resulting NDVI change curve, as depicted in the Fig. 4, illustrates a significant increase from 0.63 in 1982 to 0.68 in 2020, indicating a notable enhancement in vegetation cover over this period. An abrupt change in NDVI occurred in 2009, allowing the division of the data into two distinct periods: Stage 1 (1982–2009): The NDVI decreased from 0.63 to 0.62, exhibiting a gradual decline at a rate of −0.56 per year; Stage 2 (2010–2020): Following 2009, the NDVI increased from 0.62 to 0.68 by 2020, demonstrating a clear upward trend at a rate of 17.77 per year.

### Characteristics of spatial changes in vegetation cover

In order to better evaluate the spatial cover changes of vegetation in the area, the NDVI was partitioned with reference to the classification methods of existing studies^14^ and generally divided into bare areas (−0.3 to 0], low-coverage areas (0 to 0.2], medium-low-coverage areas (0.2 to 0.4], medium-coverage areas (0.4 to 0.6], medium-high-coverage areas (0.6 to 0.8], and high coverage areas (0.8 to 1].

**Fig. 5:**
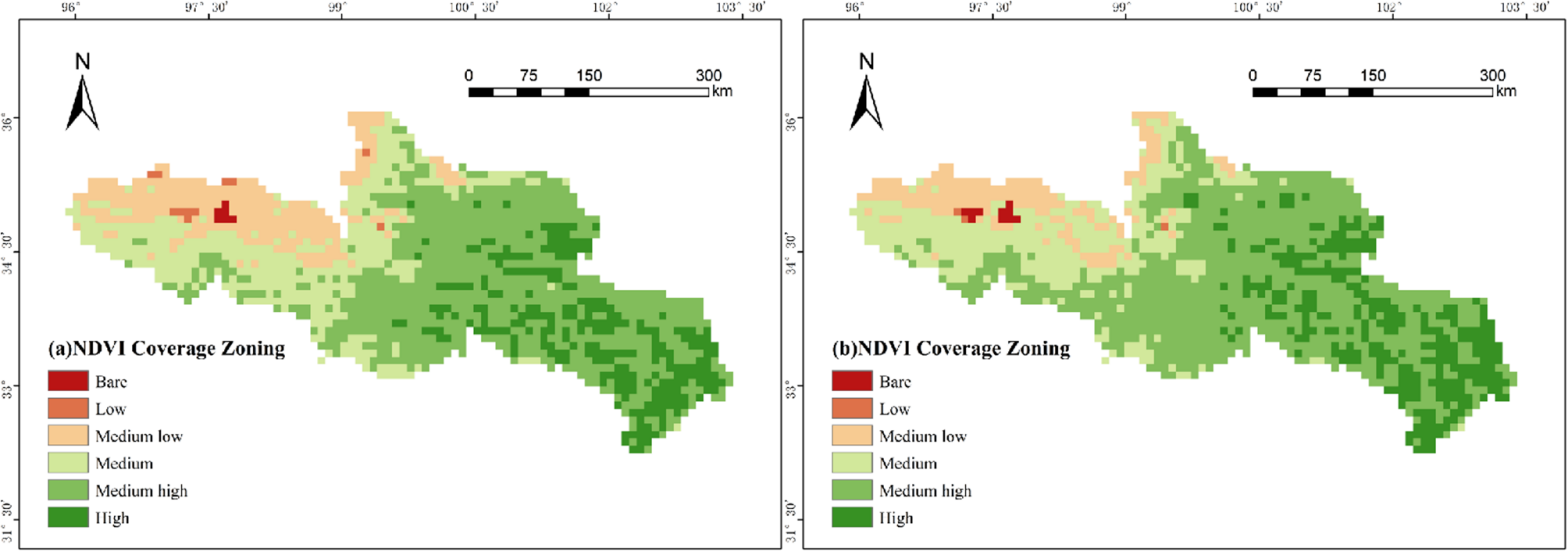
Annual average May-September NDVI coverage zones in the source region of the Yellow River (a. 1982–2009 years, b. 2010–2020 years) (Cartographic software: ArcGIS 10.6, https://desktop.arcgis.com/).

As depicted in Fig. 5, the vegetation cover in the source region of the Yellow River exhibits distinct regional variations, with generally lower coverage in the northwest and higher coverage in the southeast. In the northwestern region, encompassing Eling Lake, Zaling Lake, and the northern part of Longyangxia Reservoir, vegetation cover is sparse, resulting in lower NDVI values predominantly within the low or medium-low coverage categories. Conversely, areas such as Eling Lake, southern Zaling Lake, the northern and northeastern Bayan Kara Mountains, and the southern part of Longyangxia Reservoir exhibit better vegetation cover, with NDVI values primarily in the medium and medium-high coverage ranges. Similarly, the Ruoergai Basin and the southeastern part of the Amne Machin Mountains, among other regions, display high vegetation cover, with NDVI values predominantly in the medium and high coverage categories. In the central part of the study area, NDVI values range between 0.4 and 0.8, indicating medium to medium-high coverage. These spatial patterns align closely with the area’s altitude and climatic conditions^15^.

**Fig. 6:**
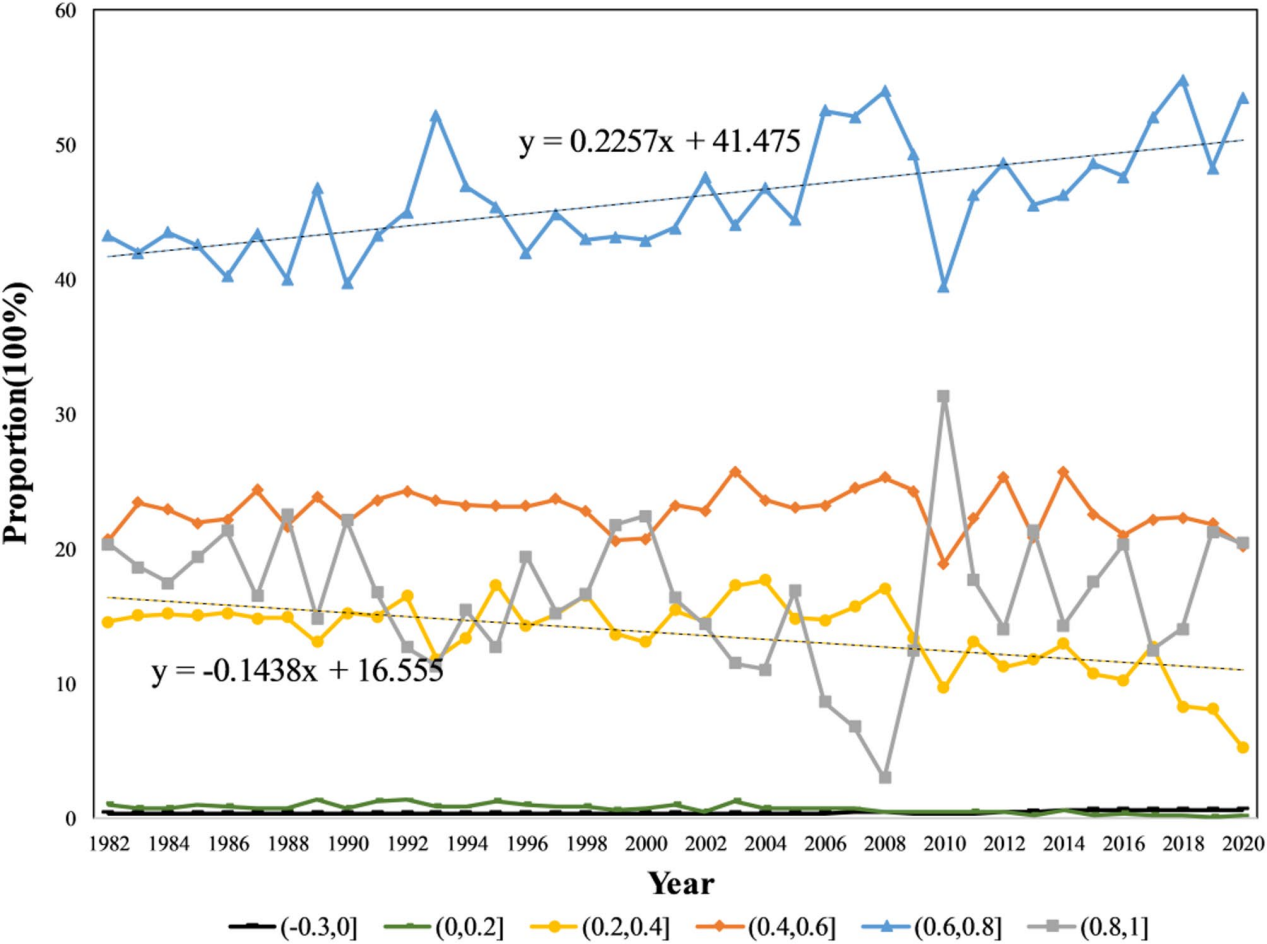
Changes in the proportion of NDVI coverage zones by year within the source region of the Yellow River.

As illustrated in Fig. 6, between 1982 and 2020, the study area exhibited relatively low proportions of exposed and low-coverage regions, with minimal significant change over time. The proportion of medium-low coverage areas decreased at a rate of − 0.1438 per year. The proportion of medium-coverage areas remained relatively stable, showing no significant change. Conversely, the proportion of medium-high coverage areas increased at a rate of 0.2257 per year, indicating notable growth in this category. The proportion of high-coverage areas fluctuated significantly, particularly around 2009: it continuously decreased before 2009, then began to rise in a fluctuating manner after 2009. Overall, since 2009, the ecological condition of vegetation in the region has been improving, with vegetation coverage gradually increasing.

### Trend analysis of vegetation cover changes

**Fig. 7:**
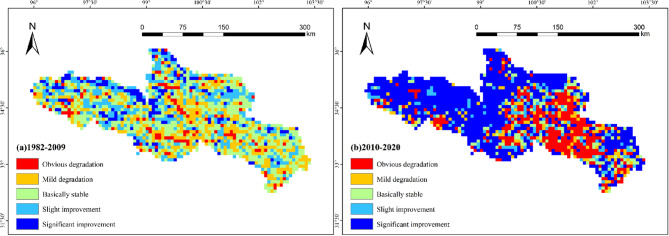
Vegetation cover change characteristics in the source region of the Yellow River from 1982 to 2020 based on Slope trend analysis (a. 1982–2009 years, b. 2010–2020 years) (Cartographic software: ArcGIS 10.6, https://desktop.arcgis.com/).

Utilizing one-dimensional linear regression analysis, the trends in vegetation cover change within the study area were examined. Figure 7 illustrates the vegetation restoration patterns during two periods: 1982–2009 and 2010–2020. Between 1982 and 2009, significant increases in NDVI were observed in the central and northern regions of the source region of the Yellow River, while vegetation coverage in most southern regions remained relatively stable. In contrast, between 2010 and 2020, the western and northern parts of the Yellow River source area showed a large-scale improvement trend. Notable vegetation enhancements were primarily concentrated in well-executed ecological restoration projects within western and northern nature reserves, such as the Eling Lake-Zhaling Lake Nature Reserve and the Zhongtie-Jungong Nature Reserve, as well as in ecological restoration pilot areas like the southwest of Dari County^15^. Conversely, regions with significant NDVI decreases were mainly located south of Zhaling Lake and Eling Lake, including areas such as Chengduo, Gande, Dari, and Jiuzhi in the southeastern part of the source area, which have also experienced vegetation degradation^16^. Overall, the vegetation cover change trend in the region demonstrates significant spatial variation. Vegetation recovery is characterized by a notable increase, with the coverage area rising from 10.15% during 1982–2009 to 55.16% in 2010–2020, marking a growth of 45.01%. Simultaneously, the proportion of lightly degraded and stable areas significantly decreased, from 24.73% to 34.06% in 1982–2009 to 7.84% and 7.78% in 2010–2020, respectively. In terms of the Slope index, the average value for 1982–2009 was − 0.000568, while it jumped to 0.001827 in 2010–2020, indicating a more evident trend of vegetation recovery.

### Impact of meteorological factors on vegetation cover

**Fig. 8:**
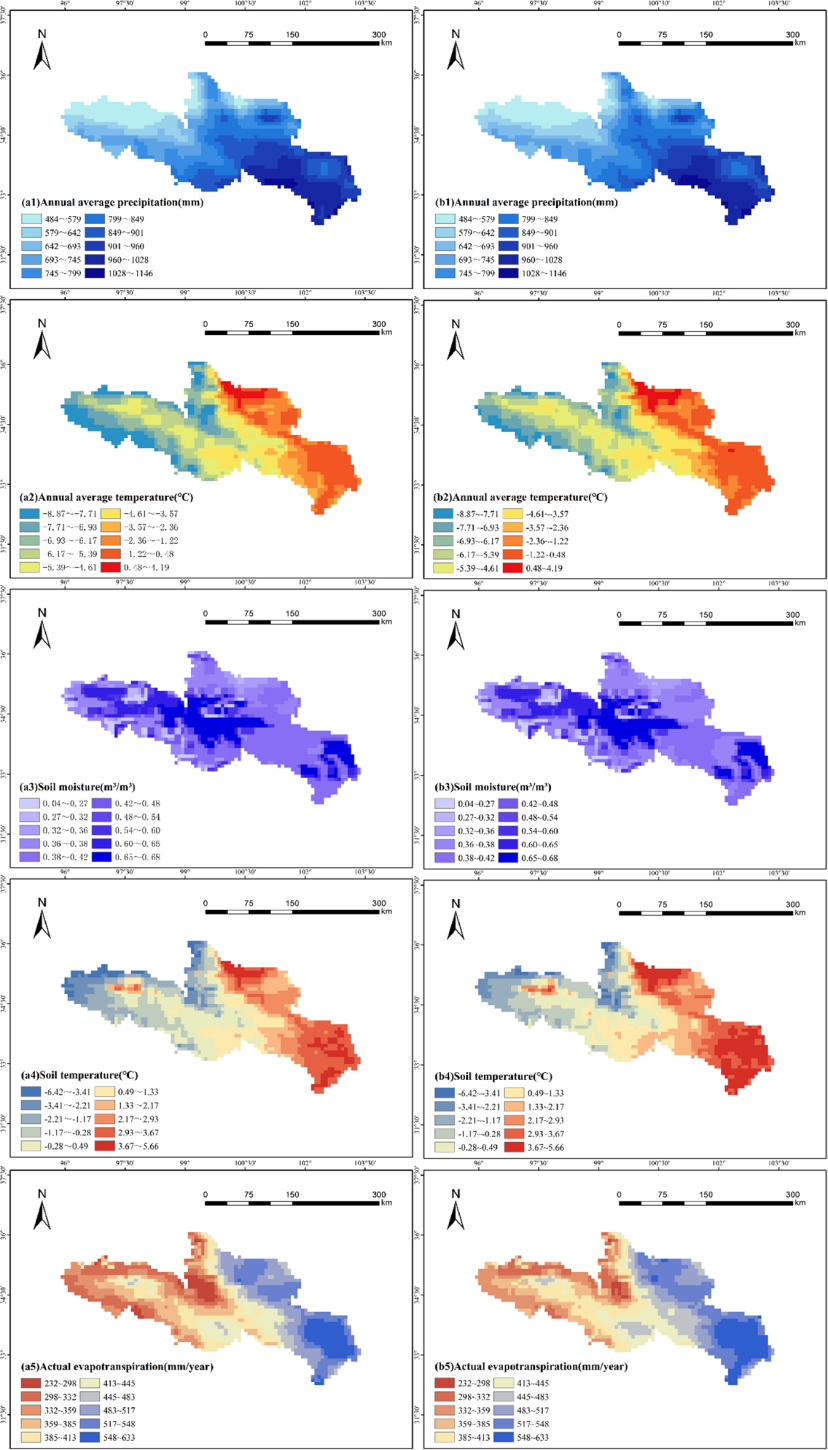
Spatial distribution of precipitation, temperature, soil moisture, soil temperature, and actual evapotranspiration in the source region of the Yellow River during 1982–2020 (a. 1982–2009 years, b. 2010–2020 years) (Cartographic software: ArcGIS 10.6, https://desktop.arcgis.com/).

As shown in Fig. 8, the average annual precipitation in the study area gradually increases from northwest to southeast, exhibiting distinct spatial differences. Between 1982 and 2009, the average annual precipitation in the upper part of the region, along Lake Eling and the Yellow River, ranged from 484 to 642 mm, while the southeastern part of the region, including Hongyuan, Ruoergai, Maqu, and Jiuzhi, received higher precipitation, with values ranging from 849 to 1146 mm. From 2010 to 2020, the average annual precipitation in the upper part of the region increased slightly, ranging from 484 to 693 mm, while it continued to rise toward the southeast, reaching 901 to 1146 mm. The mean annual temperature exhibits a clear increasing trend from west to east, with the eastern side of the source region of the Yellow River experiencing significantly higher temperatures than the western side. Between 1982 and 2020, the average annual temperatures in Tangnaihe, Maqu, and Hongyuan in the eastern part of the region ranged from − 2.36 °C to 4.19 °C, while those in Maduo, located in the western and north-central parts, ranged from − 8.87 °C to −6.17 °C. During the same period, regions with higher soil moisture were mainly concentrated in the west-central part and the southeastern Yellow River source area, including the Ruoergai Mound Plateau, the Gololok Yushu Plateau Wide Valley, and the Huangnan Mountains, where soil moisture content ranged from 0.60 to 0.68 m³/m³. Soil temperature exhibited significant spatial variation, decreasing from 2.93 °C to 5.66 °C in the Hongyuan region in the southeast to −6.42 °C to −3.41 °C in the Maduo region in the northwest. Furthermore, actual evapotranspiration in the region decreased from 517 to 633 mm/year in the east to 232 to 335 mm/year in the west.

**Fig. 9:**
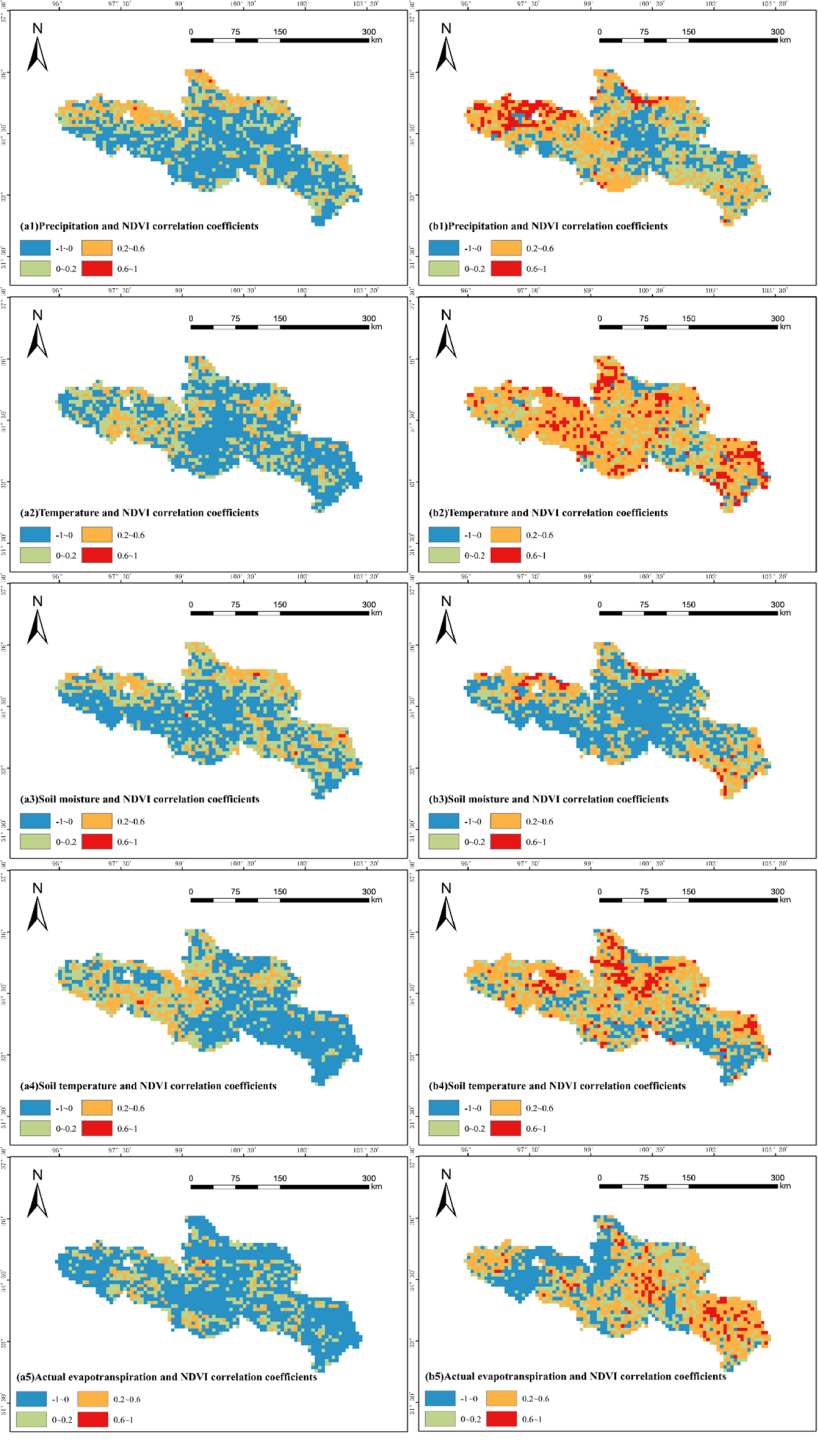
Spatial distribution of correlation coefficients between NDVI and precipitation, temperature, soil moisture, soil temperature, and actual evapotranspiration in the source region of the Yellow River from 1982 to 2020(a. 1982–2009 years, b. 2010–2020 years) (Cartographic software: ArcGIS 10.6, https://desktop.arcgis.com/).

According to the data in Fig. 9, the correlation between precipitation and NDVI in most regions during the period from 1982 to 2009 ranged from − 1 to 0.2, indicating a low correlation. In contrast, during the period from 2010 to 2020, the positive correlation coefficient between precipitation and NDVI in the source region of the Yellow River increased significantly, accounting for 72.64% of the region. The northern part of the source region of the Yellow River belongs to an arid climate zone and is adjacent to the Qaidam Basin, where water resources are scarce, temperatures are high, and the water content in the soil is insufficient. Precipitation in this region plays a critical role in promoting vegetation growth, which explains the stronger influence of precipitation on vegetation NDVI in the northern part of the source area^13^ resulting in a marked increase in the correlation between precipitation and NDVI during the period from 2010 to 2020. During 1982–2009, the correlation between temperature and NDVI was weak in most regions, with correlation coefficients mostly ranging from − 1 to 0.2. However, in 2010–2020, the correlation between temperature and NDVI improved significantly, and the correlation coefficients were positive in most regions, with values ranging from 0.2 to 1, and the positive correlation coefficients accounted for as high as 90.51%, indicating a significant increase in the correlation between temperature and NDVI.

For soil moisture, the correlation with NDVI between 1982 and 2009 was low, with little change between the two periods. Similarly, the correlation between soil temperature and NDVI was weak in 1982–2009, with most correlation coefficients ranging from − 1 to 0.2, and only 45.6% of regions showing a positive correlation. However, during the period from 2010 to 2020, the correlation between soil temperature and NDVI improved markedly, with correlation coefficients ranging from 0.2 to 1 in most areas, and 76.56% of regions showing a positive correlation.

Regarding actual evapotranspiration, the correlation with NDVI was poor during 1982–2009, with a negative correlation coefficient of 70.62%; However, during the period from 2010 to 2020, the correlation improved significantly, with 70.56% of regions showing a positive correlation, particularly in the southeastern and central areas.

In summary, the correlation between NDVI and precipitation, temperature, soil temperature, and actual evapotranspiration significantly improved during the period from 2010 to 2020. The proportion of positive correlations increased sharply. This indicates that the climate of the source region of the Yellow River shows a warming and humidifying trend with the increase of temperature and cumulative temperature, and the correlation between vegetation and meteorological factors is deepening.

To explore the degree of influence of each driver and the interaction of natural factors on vegetation cover, the weights (q-values) of the individual factors were first analyzed to identify the dominant drivers. These factors included a range of data, such as precipitation, temperature, soil moisture, soil temperature, actual evapotranspiration, land-use type, GDP, and population density.

**Fig. 10:**
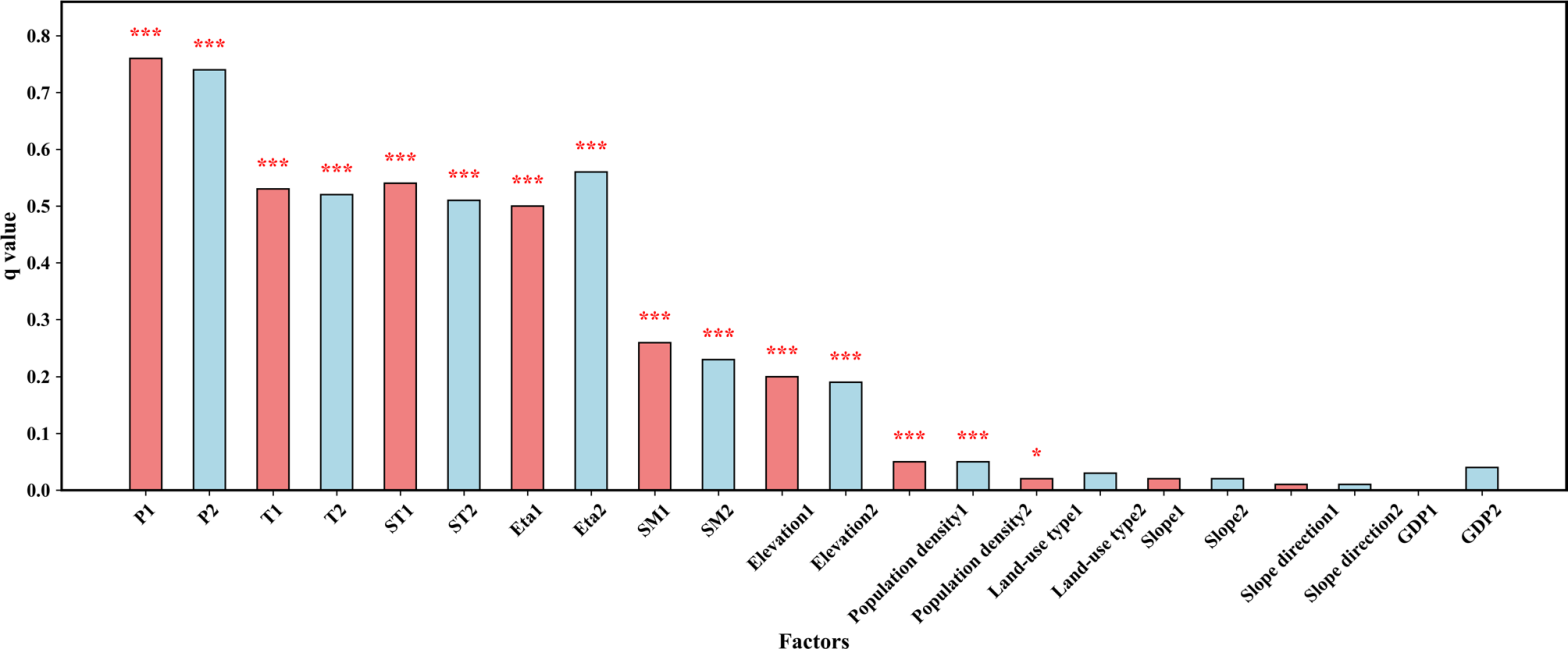
NDVI driving factor q values of vegetation in the source region of the Yellow River from 1982 to 2020 (*p* < 0.1 is represented by *, *p* < 0.05 is represented by **, *p* < 0.01 is represented by ***).

Based on the Geodetector method, the q-values of each driving factor in the region were calculated and analyzed (see Fig. 10). The results showed that the influence of each driving factor on regional NDVI during the period 1982–2009 was ranked as follows: precipitation (0.76) > soil temperature (0.55) > temperature (0.53) > actual evapotranspiration (0.51) > soil moisture (0.26) > elevation (0.18) > population density (0.05) > slope (0.02) > land-use type (0.02) > slope aspect (0.01) > GDP (0.00). And during 2010–2020, the order of influence of each driver on NDVI was: precipitation (0.74) > actual evapotranspiration (0.58) > temperature (0.52) > soil temperature (0.52) > soil moisture (0.22) > elevation (0.18) > population density (0.05) > GDP (0.04) > land-use type (0.02) > slope (0.02) > slope aspect (0.00).

It is evident that meteorological factors play a more significant role in the regional NDVI spatial differentiation. Precipitation, temperature, soil temperature, and actual evapotranspiration showed strong contributions in both time periods, with values exceeding 0.5, while natural factors such as elevation and slope, and human factors such as population density and GDP had relatively smaller contributions to NDVI. Therefore, meteorological factors are the primary drivers influencing the spatial differentiation of NDVI in the region. In both 1982–2009 and 2010–2020, precipitation remained the dominant factor influencing vegetation cover, which is closely tied to the typical arid and semi-arid climate of the source region of the Yellow River. Precipitation serves as the primary limiting factor for vegetation growth in this area.

**Fig. 11:**
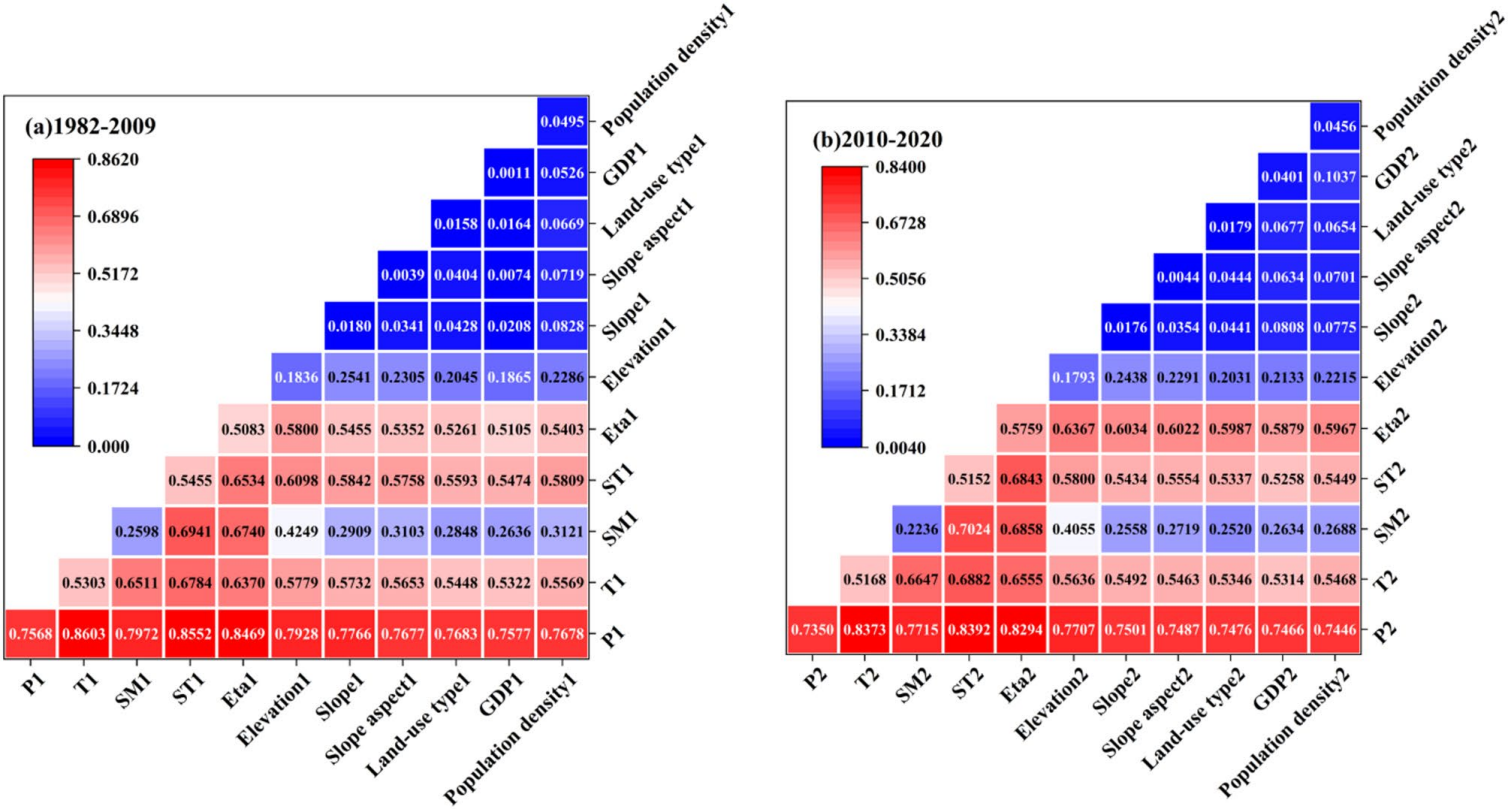
Interactive combination driving force value of NDVI driving factors in the source region of the Yellow River from 1982 to 2020 (q).

In order to focus on the variation of vegetation cover under the interaction of natural factors, the Geodetector method was used to analyze the interactions between different driving factors and their impact on the spatial differentiation of NDVI (see Fig. 11). Based on the data in Fig. 11, the following conclusions can be drawn: (1) The interaction of any two driving factors has a greater effect on NDVI than the independent effect of a single factor, showing a two-factor enhancement effect. (2) The q-values of the interaction combinations of precipitation and the rest of the driving factors are all greater than 0.7, and the q-values of the interaction combinations of temperature and the rest of the driving factors are all greater than 0.5. (3) As a whole, the changes of the regional NDVI values were significantly affected by meteorological factors such as precipitation and temperature, and the interaction combination of the driving factors had a significantly higher driving force on NDVI than that of a single factor.

Overall, the interaction of natural factors, such as precipitation and temperature, plays a crucial role in the variation of NDVI values in the region, and its effect is significantly stronger than that of meteorological or natural factors in isolation. This “enhancement effect” suggests that the factors within the ecosystem are not merely additive but are interdependent and mutually reinforcing. Climatic factors were the primary drivers in the study area, and their interactions were more complex, directly influencing the spatial distribution of NDVI.

## Discussion

The source region of the Yellow River is the core region of the Sanjiangyuan Nature Reserve and an essential water conservation zone on the Qinghai-Tibetan Plateau, where its distinctive geo-climatic environment fosters typical alpine vegetation ecosystems. As the flow-producing and water-sourcing area in the upper reaches of the Yellow River Basin, the ecosystem in this region is highly fragile and sensitive to climate change^15^. Analyzing long-term vegetation cover data to assess changes in the area is crucial for the protection and monitoring of the ecological environment, as well as for predicting future trends in vegetation dynamics.

In this study, it was found that the NDVI in the source region of the Yellow River showed an overall increasing trend during 1982–2020, and the vegetation tended to be generally favourable, which was consistent with the conclusion of Zhao Huifang et al.^24^ on the increase of vegetation area and vegetation cover in the Sanjiangyuan range from 2000 to 2019. In terms of spatial distribution, the vegetation cover gradually decreases from southeast to northwest. The areas of significant improvement in vegetation cover are mainly concentrated in the western and northern nature reserves where the ecological management project is better implemented (e.g., Eling Lake-Zaling Lake Nature Reserve and Zhongtie-Jungong Nature Reserve, etc.), as well as the pilot area of the ecological management project^13^ (southwestern part of Dari County), and seasonal permafrost areas in the southeastern part of the source area Hongyuan and Ruoergai, which are precisely the areas where the perennial permafrost degradation has been transformed into the seasonal permafrost. These areas are undergoing permafrost degradation from perennial permafrost to seasonal permafrost. Grassland degradation remains evident in the central and southern areas of Maqin and Dari, and regional ecosystem protection and restoration continue to face challenges. With a focus on these regions, the protection and restoration of the water-sourcing function of the source region of the Yellow River should be further strengthened. The trend of ecological degradation should be curbed by means of natural restoration and the implementation of major ecological restoration projects, aiming to restore key ecosystems such as alpine meadow grasslands and swampy wetlands, thereby enhancing the water-sourcing capacity. For mildly degraded areas, measures such as fencing, sealing, and comprehensive rodent control have been implemented to facilitate the natural recovery of vegetation. In areas experiencing moderate to severe degradation, artificial intervention zones have been established through black-soil-bank management combined with irrigation and grassland restoration to support vegetation recovery. Wetland hydrological recovery is facilitated through the construction of miniature check dams and marsh dredging. Centralized trampling by livestock can be reduced through the promotion of smart drinking water points, which also contribute to water conservation and improved water management in pastoral areas. Additionally, community participation can be fostered to promote ecological conservation through initiatives such as the “Livestock reduction bonus” and the appointment of “Wetland Caretakers.”

In analyzing the driving forces behind vegetation cover growth in the Yellow River Source Park, precipitation consistently emerged as the most dominant factor. This finding aligns with the conclusions of Zhao Huifang et al.^24^ who reported a strong positive correlation between vegetation cover and precipitation in the park, and with the conclusion of Zhai Dechao et al.^30^ who identified precipitation as the primary driver of productivity in alpine grasslands on the Tibetan Plateau. Precipitation contributed most significantly to aboveground net primary productivity (ANPP) during the reproductive stage (July–August) of plant development. During the wilting period (September–October), precipitation indirectly facilitated the following year’s growth by influencing the size of the shoot bank. In the dormant period (November–February), warmer temperatures helped protect overwintering buds from frost damage and indirectly enhanced the subsequent year’s productivity. Precipitation during the growing season influences the effective soil water content, while precipitation outside the growing season replenishes deep soil water reserves. In arid and semi-arid regions, the sensitivity of the precipitation–growth relationship is three to five times greater than in humid zones.

This study analyzed the spatial and temporal variations in vegetation within the source region of the Yellow River, focusing on changes in the Normalized Difference Vegetation Index (NDVI) before and after a significant shift detected in 2009, which also marked the region’s lowest NDVI value. Between 1982 and 2009, NDVI exhibited a gradual decline, whereas between 2010 and 2020, it experienced a notable increase. These findings highlight the instability of vegetation growth in the region while also indicating that ecological restoration efforts have played a crucial role in facilitating vegetation recovery.

Grasslands in the Sanjiangyuan region account for approximately 65% of the total area, and their health and growth conditions directly influence the quality of the region’s ecological environment. The pattern of grassland degradation was already established prior to the 1970 s, with degraded grasslands in Sanjiangyuan comprising 32.83% of the total area between the mid-1970s and early 1990s^25^ a trend that has persisted since. Grassland degradation in the source region of the Yellow River has become increasingly severe. The average grassland degradation rate during the 1980–1990 s doubled compared to the 1970–1980 s. As degradation progressed, pasture grasses became sparse, with significant reductions in vegetative cover and height. The proportion of high-quality forage species declined annually alongside reduced productivity, and 15–30% of the soil became desiccated and crusted, with severely affected areas developing into so-called “black soil beaches“^26^. Furthermore, desertification has intensified in recent decades, with expanding sand dunes engulfing vast areas of grassland and severely damaging land resources. In Mado County alone, desert expansion over the past two to three decades has led to the loss of extensive grasslands, forcing approximately 30–40% of herders to abandon their homes. This process is a primary driver of the persistent decline in NDVI observed up to 2009.

The initiation of ecological construction projects in the Sanjiangyuan region in 2005 had a significant impact on ecological protection efforts, initially curbing the trend of ecosystem degradation^26^. Following the implementation of these projects, ecological migration and livestock reduction measures led to a decline in the number of domestic animals, thereby alleviating grazing pressure on grasslands. The “returning livestock to grassland” initiative further reduced livestock numbers in project areas, lowering actual grazing intensity and partially mitigating grassland degradation. Management of degraded grasslands in black soil areas, along with rodent pest control, has contributed to restoring these ecosystems and preventing further degradation. Reforestation efforts involving mountain closures and wetland forest protection have increased forest coverage, canopy density, and biomass storage. Wetland conservation initiatives have helped mitigate human disturbances on wetland ecosystems. Measures such as ecological migration, improved livestock management, and solar energy utilization have contributed to reducing human disturbances in nature reserves and lowering land use intensity. As a result, the influence of human activity on ecosystems has diminished, localized improvements in ecological structure have been achieved, the pace of structural change has slowed, grassland degradation has been partially mitigated, and the expansion of desertification has been significantly curbed or even reversed.

In recent years, the statistical characteristics of climatic elements in the source region of the Yellow River have exhibited increasingly pronounced changes. Hao Zhenchun et al.^27^ reported a significant warming trend in the source area, with a temperature increase of 0.31 °C per decade, alongside a modest upward trend in precipitation, indicating a shift toward warmer and more humid conditions. Similarly, Bai Luyao et al.^28^ observed significant interannual temperature fluctuations, with an overall warming rate of 0.37 °C per decade and a clear trend of increasing precipitation since the late 20th century. Qiao Shijiao et al.^29^ further found that the average annual temperature, as well as extreme high and low temperatures across meteorological stations in the region, exhibited consistently significant upward trends. These findings collectively indicate a warming and humidifying climate, with increased precipitation and rising temperatures contributing to greater glacier meltwater, which in turn supports ecosystem recovery. Following the implementation of ecological projects, the average annual temperature at meteorological stations in the Sanjiangyuan area rose by 1.29 °C compared with the 1975–2004 period, while average annual precipitation increased by 61 mm. The elevated temperature advanced the onset of vegetation rejuvenation, thereby enhancing vegetation productivity. Glacial retreat and increased freeze–thaw activity elevated the volume of glacial and permafrost meltwater in the watershed, which not only fostered vegetation recovery but also supported broader ecosystem restoration and improved the seasonal distribution of runoff. The increase in precipitation can be attributed both to natural climate variability and to artificial rainfall augmentation implemented through ecological projects. Between 2006 and 2009, such efforts contributed an additional 26.066 billion m³ of precipitation, primarily in the Yellow River Basin. The combined effects of rising temperature and increased precipitation have significantly enhanced the region’s warming and humidification trend, leading to slowed desertification, reduced desert area, expanded water bodies, increased grassland productivity, and a rise in the theoretical livestock carrying capacity^26^. The implementation of ecological protection projects may have influenced local climate conditions, contributing to the accelerated growth of vegetation cover and potentially explaining the observed NDVI minimum and abrupt shift in 2009. The responsiveness of NDVI to meteorological conditions underscores the critical role of climate as a primary driver of vegetation dynamics and a key factor in enhancing vegetation cover in the source region.

## Conclusion

The aim of this study was to investigate the spatial and temporal evolution of vegetation cover and the trend of NDVI in the source region of the Yellow River from 1982 to 2020. Additionally, the study analyzed the influence of various driving factors on NDVI by integrating natural factors with one-way linear regression, correlation analysis, and the geo-detector method for a comprehensive assessment. Overall, meteorological factors had a significant impact on the spatial and temporal patterns of vegetation cover in the region. The main findings are summarized as follows:

1. During the period of 1982–2020, NDVI in the source region of the Yellow River exhibited a significant upward trend; however, an abrupt change occurred in 2009, leading to a subdivision into two distinct phases. During Phase 1 (1982–2009), NDVI experienced a slight decline at a rate of −0.56∙a⁻¹, whereas in Phase 2 (2010–2020), it increased markedly, with a growth rate of 17.77∙a⁻¹. The shift in NDVI growth observed around 2009 was closely associated with regional climate warming and the implementation of ecological and environmental protection policies;

2) The distribution pattern of NDVI in the source region of the Yellow River shows a high value in the southeast and a low value in the northwest, with a gradual increase from northwest to southeast, exhibiting significant spatial variation. Vegetation cover is sparse in the northwest regions, such as the areas around Erling Lake, Zhaling Lake, and the northern part of Longyangxia Reservoir, while vegetation cover is better in the southern parts of Erling Lake, Zhaling Lake, the northern part of Bayan Har Mountain, and the southern part of Longyangxia Reservoir. Higher vegetation coverage is also observed in areas like the Ruoergai Basin and the southeastern part of the Animaqing Mountain;

3) The source region of the Yellow River is characterized by a typical arid and semi-arid climate, and precipitation is the primary limiting factor for vegetation growth. Geodetector-based analysis indicates that precipitation consistently serves as the dominant driver of vegetation growth in the source region of the Yellow River, and that NDVI variations are significantly influenced by meteorological factors, particularly precipitation and temperature. Moreover, the interactive effects among driving factors exhibit significantly stronger explanatory power than individual factors, demonstrating a “two-factor enhancement effect.” In future vegetation restoration efforts, greater attention should be directed toward the climate–vegetation feedback mechanism, with comprehensive consideration of the dynamic balance between water resources and vegetation.

This study reveals the spatiotemporal evolution patterns and driving mechanisms of NDVI in the source region of the Yellow River; however, ensuring the long-term sustainability of vegetation restoration necessitates multidisciplinary collaboration. Future recommendations include:1) Analyze multifactor nonlinear interactions using machine learning techniques (e.g., SHAP analysis), with an emphasis on identifying precipitation–temperature thresholds that lead to abrupt NDVI changes; 2) Develop an integrated permafrost–vegetation–hydrology model using high-resolution remote sensing and in-situ observational data; 3) Optimize vegetation restoration strategies according to ecological zoning, for example through integrated approaches combining wetland dredging and replanting with cold-tolerant grasses; 4) Integrate NDVI dynamics into the ecological security assessment framework to support evidence-based planning for the high-quality development of the Yellow River Basin.

## Data Availability

The datasets used and/or analyzed during the current study available from the corresponding author on reasonable request.
